# Patient Portal Use and Experience Among Older Adults: Systematic Review

**DOI:** 10.2196/medinform.8092

**Published:** 2017-10-16

**Authors:** Dawn K Sakaguchi-Tang, Alyssa L Bosold, Yong K Choi, Anne M Turner

**Affiliations:** ^1^ Department of Human Centered Design and Engineering College of Engineering University of Washington Seattle, WA United States; ^2^ Department of Health Services School of Public Health University of Washington Seattle, WA United States; ^3^ Department of Biomedical Informatics and Medical Education School of Medicine University of Washington Seattle, WA United States; ^4^ Biomedical Informatics and Medical Education University of Washington Seattle, WA United States

**Keywords:** aged, patient portals, personal health records, utilization, usability, user experience

## Abstract

**Background:**

The older adult population (65 years or older) in the United States is growing, and it is important for communities to consider ways to support the aging population. Patient portals and electronic personal health records (ePHRs) are technologies that could better serve populations with the highest health care needs, such as older adults.

**Objective:**

The aim of this study was to assess the existing research landscape related to patient portal and ePHR use and experience among older adults and to understand the benefits and barriers to older adults’ use and adoption of patient portals and ePHRs.

**Methods:**

We searched six pertinent bibliographic databases for papers, published from 2006 to 2016 and written in English, that focused on adults 60 years or older and their use of or experience with patient portals or ePHRs. We adapted preferred reporting items for systematic reviews and meta-analyses (PRISMA) guidelines to review papers based on exclusion and inclusion criteria. We then applied thematic analysis to identify key themes around use, experience, and adoption.

**Results:**

We retrieved 199 papers after an initial screening and removal of duplicate papers. Then we applied an inclusion and exclusion criteria, resulting in a final set of 17 papers that focused on 15 separate projects. The majority of papers described studies involving qualitative research, including interviews and focus groups. They looked at the experience and use of ePHRs and patient portals. Overall, we found 2 main barriers to use: (1) privacy and security and (2) access to and ability to use technology and the Internet. We found 2 facilitators: (1) technical assistance and (2) family and provider advice. We also reported on older adults’ experience, including satisfaction with the system and improvement of the quality of their health care. Several studies captured features that older adults wanted from these systems such as further assistance managing health-related tasks and contextual health advice and tips.

**Conclusions:**

More research is needed to better understand the patient portal experience among older adults from initial use to adoption. There are also opportunities to explore the role of design in addressing barriers and supporting facilitators to patient portal and ePHR use. Finally, the future use of these systems by older adults should be anticipated and considered in the design process.

## Introduction

### Background

In 2014, the adult population aged 65 years or older in the United States was 46.2 million, and this number is projected to increase to 98 million by 2060 [1]. With this expected growth in the older adult population, it is essential for communities to consider ways to support their aging population. To this end, there has been a growing interest in the design of technologies for older adults, including technologies that can support older adults through health maintenance and health information management. Such technologies have the potential to support older adults by allowing them to age in their own homes, maintain their health, and provide a sense of autonomy. Although there have been gains in the adoption of technology by adults 65 years or older, older adults have consistently trailed the general American population, especially in adopting digital health technologies [2,3].

Interest in electronic health records (EHRs), patient portals, and electronic personal health records (ePHRs) has increased in recent years [4-6]. Ancker et al (2016) [6] conducted a survey of New York State residents to understand the rate of patient portal and personal health records (PHRs) adoption over time. They found that use of PHRs by New Yorkers increased from 11% in 2012 to 27.1% in 2015. Ford et al (2016) [5] forecasted the adoption of PHRs based on the 2008, 2011, and 2013 Health Information National Trends Surveys. They anticipated that PHR adoption will grow beyond 75% by 2020. These studies show that the use of patient portals and PHRs will likely continue to grow.

The level of research on this topic raises awareness about how these technologies are being used and has implications for improved and innovative design. Research on digital health technology adoption by older adults also signals a focus on how technology could better serve populations with the highest needs, who often manage complex health conditions and multiple chronic illnesses. The incidence of multiple chronic conditions increases with age [7], and the prevalence of some chronic conditions such as hypertension, asthma, cancer, and diabetes has also increased among older adults [8]. Hospitals, clinics, and organizations have started to offer patients a way to stay connected to their health information and manage their wellness and health care needs through patient portals and ePHRs.

There are several definitions of ePHRs and patient portals within the literature, and patient portals are sometimes described as a type of ePHR. For the purposes of this paper, *patient portals* are defined as systems for health information management that are linked, or tethered, to a patient’s EHR [9,10]. For example, the US Department of Veterans Affairs offers patients access to My Health *e* Vet [11], and several hospitals in the United States use Epic’s MyChart portal [12]. Both portals give patients access to their health information and include features such as the ability to schedule appointments, view test results, request prescription renewals, and send messages to health care providers. In addition to tethered patient portals, there are *ePHRs* that are not connected to EHRs, such as Microsoft HealthVault and the Health app on Apple devices. In these systems, the individual is responsible for entering their own health information. ePHR systems often include features such as health tracking or medication lists. Other features of these systems include the ability to share health information with others and track fitness and personal health goals. The major distinction between ePHRs and patient portals is that patient portals are tethered (to EHRs) and ePHRs are not. Both offer a centralized location for storing and organizing electronic health information.

### Objectives

Although much has been written about the use of patient portals and ePHRs in general, there is less material focused on the use of patient portals by older adults. Technologies such as patient portals and ePHRs have the potential to help older adults by strengthening their ability to manage, understand, and control their health information. However, it is a leap to assume that patient portals and ePHRs, as they are currently designed and used, will effectively address the health information needs of the older adult population. It is important to first understand the facilitators of and barriers to older adult use and adoption of health-related technology. It is also important to understand their experiences with ePHRs and patient portals and how these experiences have influenced or changed their personal health information management. Understanding the facilitators and barriers will provide insights to why older adults decide to use or adopt patient portals and ePHRs. Similarly, learning about older adults’ experiences with these systems and their impact on health information management can provide guidance on how to improve their design and ensure their effective use and adoption. Finally, it is important to understand what design recommendations have been proposed, and what is important to older adults. Considering these objectives, the goal of this systematic review was to investigate the existing research landscape with a focus on answering the following questions:

1. In the literature, what barriers and facilitators to older adults’ use and adoption of patient portals and ePHRs have been described? What is the evidence that these barriers and facilitators exist?
2. How do older adults describe their experience using patient portals and ePHRs?
3. What design recommendations have been proposed to help overcome barriers and enhance facilitators of older adults’ experience, use, and adoption of patient portals or ePHRs?

## Methods

### Revised PRISMA protocol

We adapted the preferred reporting items for systematic reviews and meta-analyses (PRISMA) 2009 checklist to guide our systematic review of the use of patient portals and ePHRs among older adults [13]. As PRISMA is positioned toward standardized study designs, such as clinical trials that aim to support universal interpretation of results, we modified the PRISMA protocol to accommodate the study methodologies in this review more common to information sciences, specifically qualitative and mixed-method studies. Thus, we reviewed the methods and metrics used in the studies rather than the standardized outcome variables one would typically see in traditional systematic reviews of controlled trials. Our protocol included a systematic search, a study selection, and a qualitative review of the findings.

### Literature Search

We conducted our search in six databases that spanned the medical, nursing, and engineering literature. These databases were PubMed, EMBASE, CINAHL Complete, Compendex (includes ACM digital library and IEEE XPlore), and Inspec. We consulted with librarians in the University of Washington Health Sciences and Engineering libraries on the selection of databases and the mechanics of using them (eg, controlled vocabulary, using filters, and syntax). We also received assistance narrowing down keywords to use. We searched all databases with the keywords “older adult,” “seniors,” or “elders,” and “patient portal,” “electronic medical record,” or “personal health record” (see Table 1). We did a general search in Google Scholar to find potential papers that did not result from our searches in the other databases. In PubMed and EMBASE, we used additional keywords such as usage, utilization, adoption, and patient satisfaction. We did not use the additional keywords in CINAHL Complete, Compendex, and Inspec because it narrowed rather than broadened our search results. We limited our search to papers published within a 10-year period (January 2006-November 2016). Although we recognize that a 10-year period is a broad timeline given the fast pace of advancement in technology, we selected this time range to get an expanded view about needs and experiences of older adults related to health information technology, and included commentary on changes in technology and findings over time.

**Table 1:** Searches used in each database.

Database	Description	Citations
PubMed	older adult OR seniors OR elderly OR aged AND patient portal OR electronic health record OR personal medical record OR personal health record AND usage OR using OR utilization OR utilize OR adopt OR adoption OR preferences OR patient access to records OR patient satisfaction AND english NOT letter OR editorial AND last 10 years	885
EMBASE	older adult OR older adults OR seniors OR elderly OR aged OR aged AND patient portal OR electronic medical record OR personal medical record OR personal health record AND usage OR utilization OR utilize OR adopt OR adoption OR preference OR patient access to records OR patient attitude AND english AND [embase]/lim NOT [medline]/lim AND (2006-2016)/py	409
CINAHL	patient portal OR electronic health record OR personal health record older adult OR senior OR elder Limiters: published date: 2006-01-01-2016-12-31; English language	129
Compendex and Inspec	older adult OR senior OR elder AND electronic health record OR personal health record AND 2006-2016 AND english	484

**Inclusion criteria.**

Inclusion criteria

- Include participation of adults who were 60 years or older. These older adults could be the sole focus of the study or be a group of adults who were part of a larger study. Typically, older adults are characterized as 65 years and older; however, we decided to use a wider age range to include a broader set of papers.
- Focus on patient portals or ePHRs
- Discuss use, adoption, or experience with patient portals and ePHRs or features of those systems (eg, studies that evaluated patient experiences using secure messaging with providers, or having electronic access to medical records)
- Examine features of patient portals and ePHRs to inform design
- Published from 2006 to 2016
- Written in English

### Inclusion and Exclusion Criteria

Papers were selected based on the inclusion criteria in (Textbox 1) and exclusion criteria provided here. We excluded studies that were not focused on older adults’ use, experience, or adoption of patient portals, ePHRs, or features of those systems. Although studies do not consistently report clear definitions of use and adoption, we chose to differentiate between these two terms for this review. Specifically, we refer to *use* as short-term activity within a patient portal for a period of less than 1 year, whereas we define *adoption* as a commitment to continued use of systems beyond 1 year. We defined *experience* as a person’s perceptions of their interactions with patient portals or ePHRs. We also included *formative* studies that were focused on information gathering for design, including user testing of new systems and assessments to inform development of systems or test the acceptability of particular systems. Formative studies were not focused on adoption or use or factors influencing the initial use of a particular developed system. The types of papers that were excluded were studies focused on patient online communities or the provider experience using patient portals or ePHRs. Papers that solely recorded log-in data and demographics were also not considered to be focused on use and were excluded from this review. In addition, we excluded nonempirical studies such as commentaries, letters to the editors, notes, books, reviews, and conceptual papers.

One researcher (DST) conducted an initial screening of the paper titles and abstracts, removing records that were irrelevant such as those focused on provider experience, implementation of EHRs, and using EHRs to recruit participants. Then 3 researchers applied the inclusion and exclusion criteria to the abstract of each paper using the Covidence (Melbourne, Victoria) software [14]. Each paper was reviewed by at least 2 of the 3 researchers (DST, AB, and YC), and any disagreements were discussed. In cases where a resolution could not be reached, a third researcher made the final decision. After excluding an initial set of papers, the same 3 researchers applied the inclusion and exclusion criteria to the full text of the papers using the same process described above to resolve disagreements.

After applying the inclusion and exclusion criteria, we conducted a thematic analysis of the papers. Two researchers (DST and AB) created codes using an inductive process. They summarized each of the papers and collectively came up with a list of key points from the summaries and from the papers themselves. These key points were then grouped into codes. The groups of codes were then further refined into themes, and the final list of themes was informed by the project’s research questions and decided collectively in a meeting with the team researchers.

### Quality Review

We reviewed the papers using the top two guidelines from the mini Statement on the Reporting of Evaluation studies in Health Informatics (STARE-HI), ranked as essential by professionals in health informatics for reporting studies [15]. They were “Interpret the data and an answer to the study question” and “Description of the outcome measure or evaluation criteria.” We added two additional guidelines because they provided key information related to our study questions: “Provides a description of system and its functionalities” [16] and “Provides clear description of how results impact design recommendations.” We gave the papers a score for each of the four guidelines outlined above. The score ranged between 0 (does not meet the criteria) and 2 (fully meets the criteria), for a total score of 8.

## Results

**Figure 1:**
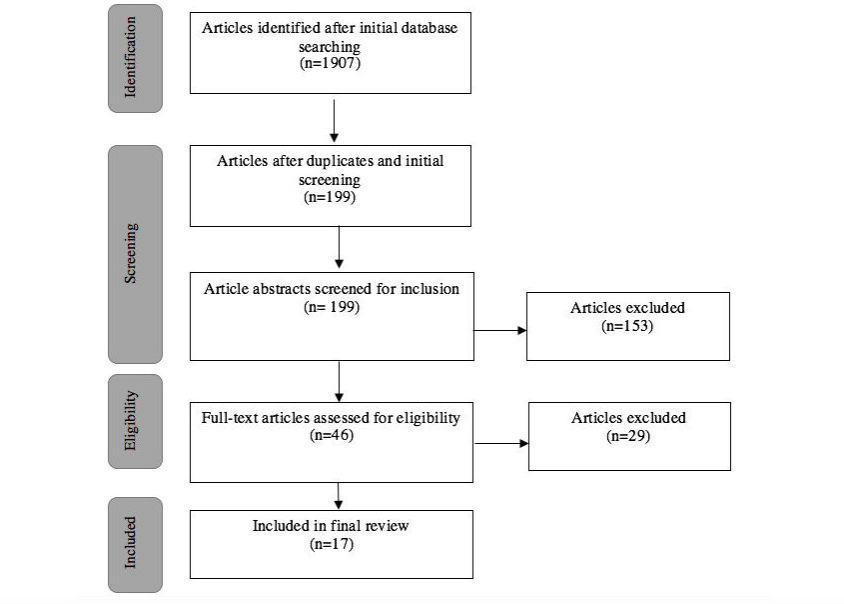
Systematic review process.

The search returned 1907 papers in total after removing duplicates. An initial screening of paper titles and abstracts resulted in 199 papers. Abstract review, described above, resulted in 46 papers for full-text review. The full-text review resulted in a final set of 17 papers (see Figure 1).

### Description of Papers

The final set of 17 papers focused on 15 separate projects (see Multimedia Appendix 1). Papers spanned the 10-year period from 2006 to 2016. All papers published before 2014 examined ePHRs, whereas those papers published from 2014 to 2016, with the exception of one [17], looked at patient portals. Of the 17 papers, 7 (41%) were conference proceedings. All conference proceedings were peer-reviewed. Authors used a range of research methods in the final set of papers: 10 of 17 (59%) were interviews, observations, focus groups, design sessions, and user studies; 9 of 17 (53%) were surveys or questionnaires; and 4 of 17 (24%) were mixed-methods studies. The sample size of the papers ranged from 16 participants in a user study to 231,082 participants in a survey. Six papers focused on patient portals [9,10,18-21], 8 papers focused on ePHRs [22-29], and 2 papers looked at other similar systems, specifically a personal health application and the Swedish medication registry [30,31]. Half (8/17) of the papers evaluated patient portals or ePHR systems overall [9,18-20,23,24,26,29]; others focused on specific features such as messaging systems [25] or medication management tools [22,23,30,31].

Seven papers focused on short-term use or factors influencing the initial use of a system. Nine papers were primarily formative, collecting information related to system design, development, or usability. Formative papers collected information to inform design of systems generally [9,10,20,21,28,30] or focused on developing specific systems [18,22,23]. Only 3 papers compared short-term use and long-term adoption [17,24,25]. In 2 cross-sectional papers, Lam et al (2013) [25] and Zettel-Watson and Tsukerman (2016) [17], participants most commonly reported using systems anywhere from 1 month to 1 year and reported an average period of use of over 3 years, respectively. In the Kim et al (2009) [24] paper that looked at patterns of use longitudinally, 51% of the participants only used the system once during the first year of the study period.

Qualitative and cross-sectional papers provided insight into both specific systems and general experience. In 3 of the 17 papers (18%), participants used a system and were given a survey or questionnaire to gain feedback on their experience [19,25,31]. There were 2 papers (12%) that evaluated a system in a lab setting [9,18] and 3 papers, focused on two projects, (18%) [24,26] where participants used a system in a community setting such as a retirement or housing facility [22,24,26]. Four papers (24%,) did not focus on a specific system but instead asked participants to reflect on their experiences with patient portals or ePHRs in general [10,17,21,27]. Two papers (12%, 2/17) focused on developing a personal health application with participants [23,30]. Another approach that 3 papers (18%, 3/17) took was to gather information needs from participants through qualitative methods such as interviews, design sessions, and a diary method to inform design of a system [21,22,28] (see Multimedia Appendix 2).

### Participant Characteristics

Demographic details about participants are provided in Multimedia Appendix 2. All papers had participants who were 65 years or older [9,10,17-31]. Two papers [19,31] analyzed differences between age categories within the older adult group. In all other papers, the older adults were reported as one group. Of the 13 studies that reported gender, 11 had more female than male participants [9,10,17,19-21,24,26,27,30,31].

### Quality Review

All of the papers met the criteria “Interpret the data and an answer to the study question,” and almost all (14 of 17) met the criteria “Description of the outcome measure or evaluation criteria.” The last two criteria were more varied. There were 7 papers that did not provide enough detail about a system and its functionalities [17-19,21,22,29,30]. For example, Sack et al (2011) [29] evaluated PHR technologies using Web and mobile-based Google Health. In discussing the technologies, they did not provide details about the features or functions of the system beyond it being Web or mobile-based. Papers were given full points if they provided a definition including functionality for a patient portal or an ePHR. Descriptions of the system provide a context for results and recommendations. It also provides a status of the technology at that time.

The other criterion that papers were varied on was “Provide clear description of how results impact design recommendations.” Although a majority of papers did not aim to provide design recommendations, one of our research questions was to learn about design recommendations that have been proposed to address the barriers and facilitators to use, adoption, and experience. We did find that 15 of 17 papers connected their findings to design considerations or suggestions for improving use of system [9,10,17-23,25,27-31], for example, training to increase adoption [27]. Papers were given a partial score if their recommendations were brief and vague. Papers received full points if authors offered clear considerations for design and gave detailed recommendations. For detailed ratings, see Multimedia Appendix 3.

### Barriers

We found commonalities among all papers concerning barriers and facilitators to the use and adoption of patient portals or ePHRs by older adults. We identified two main barriers across studies: (1) privacy and security and (2) access and ability to use technology and the Internet.

#### Privacy and Security

In 7 papers, older adults expressed a concern about the privacy and security of their information when using patient portals, ePHRs, or Web-based health management tools [10,17, 20-22,26,28]. Privacy and security concerns were linked to the storage and use of data collected in patient portals. Hourcade et al (2011) [22] reported that participants were worried about pharmaceutical or drug companies accessing and misusing their data. Despite reassurance that the research was confidential and for academic purposes, participants expressed worry that researchers might not fully disclose partnerships with government institutions or drug companies. In the Kerai et al (2014) [20] paper, 63% of participants were concerned about security. Participants in the Latulipe et al (2015) [21] paper were concerned that the government or insurance companies would access their records without their permission. In the Lober et al (2006) [26] paper, participants were living in a government housing authority and had to be able to live independently to stay there. They were protective of their health information because they did not want to be evicted if their physical health limited their ability to remain independent.

#### Access and Abilities

Lack of access to technology and the Internet was mentioned as a barrier in 5 papers. However, the results from these papers are based on small sample sizes, and two of them were focused on lower income communities. In the papers we reviewed, disparities in age, race, and ability to pay for the Internet were mentioned. Turner et al (2015) [10] reported that some of their participants had difficulty accessing the Internet because of its cost. Logue and Effken (2012) [27] identified that older and younger seniors had similar access to computers but differed in Internet access. Seniors over the median age of 78 years had less access to and familiarity with the Internet than seniors aged under 78 years [27]. Of the 38 participants in the Lober et al (2006) [26] study, 27 (71%) did not own computers. Latulipe et al (2015) [21] reported that older adults were aware of Internet access in their communities, and over half had a digital device such as a computer, laptop, or tablet. However, some participants did not have access to the Internet at home, suggesting that the devices were not being used [21]. Two papers noted gendered differences in Internet access, but results were mixed [20,27].

Seven papers defined computer and Internet skills as a barrier, and papers focused on both actual and perceived abilities. Lober et al (2006) [26] reported that major barriers to use of their portal system were computer literacy and computer anxiety. They described computer literacy as instances where participants were unable to do tasks on their own, such as turning on the computer or using a mouse or keyboard. Computer anxiety was a refusal to complete tasks on the computer, despite having the cognitive or physical abilities to accomplish the tasks. Turner et al (2015) [10] also identified that confidence in the ability to use computers and computer anxiety impacted the use of patient portals. Turner et al (2015) [10] found that of the 59 participants who were nonusers of patient portals, 19% (11/59) had never learned how to use a computer [10].

Disparities in age and race were also mentioned. Logue and Effken [23] found that older seniors were less confident than younger seniors in their ability to use an Internet-based PHR. Older seniors (older than 78 years) were also less likely to know how to find health resources on the Internet and less interested in using PHRs [23]. Gordon and Hornbrook (2016) reported that 10.09% (260/2602) of seniors surveyed received help from someone to go on the Web or had someone go on the Web for them. They also found Chinese, non-Hispanic whites, and younger seniors (aged 65-69 years) were more likely to use the Internet for email and health-related tasks than black, Latino, and Filipino seniors and those who were aged 75 years and older [19].

Some studies also mentioned disparities based on physical and cognitive ability [19,26]. Lober et al (2006) [26] found that 13 of 38 participants had cognitive issues that impacted their use of a computer, presenting problems specifically when remembering the URL of the system, usernames, and passwords. Older adults with vision, hearing, and physical limitations leading to decreased mobility had difficulty using the system on their own [26]. Gordon and Hornbrook (2016) [19] also reported that physical issues inhibit use of a computer or the Internet. They noted that this posed more of a problem to seniors in the oldest age group (75-79 years) [19].

### Facilitators

We identified two major factors that facilitated older adults’ use and adoption of patient portals and ePHRs: (1) technical assistance and (2) the advice of family and providers.

#### Technical Assistance

Three papers mentioned the role of technical assistance in initially facilitating portal use [19,22,24]. Hourcade et al (2011) [22] described a video to help present the ePHR that they were testing among older adults. They also explained that they saw a benefit in working with older adults over several weeks, which allowed them to introduce older adults to the ePHR concept, assist with system navigation, and ultimately gather more meaningful feedback from a group that was informed about the ePHR tool [22]. In their paper, Gordon and Hornbrook (2016) [19] found that participants wanted technical assistance with using a portal and preferred help from a person rather than a Web video [19]. Kim et al (2009) [24] had graduate nursing students available to assist participants with using a patient health information management system (PHIMS) portal. They noted that the most frequent use of PHIMS coincided with the days when the nursing students were onsite [24].

#### Family and Provider Advice

Other papers noted family and provider advice as facilitators to portal use. Lam et al [25] found that participants were significantly more likely to be introduced to a portal messaging system by their providers than were nonusers [25]. Similarly, Zettel-Watson and Tsukerman (2016) [17] reported that patients cited their doctor’s recommendation as being important when initially using the portal but not for adoption or continued use. Logue and Effken (2012) [27] found that Hispanic women, in particular, were likely to be influenced to use a PHR based on a family member’s recommendation. Forty-six percent of Hispanic women stated that this was the case. They also reported that older adults who felt they were a part of a team with their health care provider were more motivated to try a PHR, to believe that an Internet-based PHR would give them their desired health outcomes, and to select a particular practice because PHRs were a part of care [27].

### User Experience

The papers that were reviewed spanned a 10-year period. This is considerable, as technology tends to rapidly change over time. It is likely that experiences with newer technologies are different from older technologies. In the papers that we reviewed, we found 10 papers from 2006 to 2013 that focused on ePHRs, whereas 6 papers from 2014 to 2016 focused on patient portals.

There were several papers that evaluated participants’ use of patient portals, ePHRs, or Web-based health management tools [9,10,17-21,24,26,27,29,31]. Participants reported an overall satisfaction with the system they used [25,24]. In addition to participants’ satisfaction with the system, they reported that the system was useful and it improved the quality of the health care they received [24,26]. Sack et al (2011) [29] conducted focus groups to evaluate mobile PHRs versus Web-based PHRs. They used a cost (negative comments) versus benefit (positive comments) analysis as a strategy to interpret their findings. They found that overall there were more benefit comments than cost comments for Web-based PHRs [29].

#### Function and Usability

Although some studies reported an ease of use in setting up and accessing their accounts, transferring information, and navigating the system [10,17], there were also some studies that raised usability issues such as difficulty in logging in and navigating a complex system [10,21]. These issues can negatively impact the user’s experience and may interfere with a user’s ability to complete tasks. Lam et al (2013) [25] found that some of the participants (19.0%) (31/163) who logged into a patient-physician messaging system wanted added features and functionality, 11.0% (18/163) wanted more providers in the system, and 4.3% (7/163) wanted faster response to messages [25]. Khan et al (2010) [23] found participants appreciated pictorial representations on the Colorado Care Tablet interface but had difficulty understanding what they represented. They suggested adding text to describe the pictures [23]. Two papers found that participants did not like entering text information and preferred the system to do more data input [22,23].

#### Features

In several papers, participants reported features of systems that they frequently used and liked. They appreciated the health information management tasks such as checking lab results, learning about health conditions [17], preparing for appointments through medication list management [23,31], and record management [17]. Participants also appreciated the ability to communicate directly with providers through secure messaging [10,19].

Six papers identified features that participants wanted from a patient portal or an ePHR system. Two mentioned that participants wanted to share health information, such as medication lists, with others or share different views of their health information depending on the person or situation [28,30]. Participants in the Sack et al (2011) [29] paper suggested that medical personnel should have a security password for record access in emergency situations.

Several papers indicated participants’ desire for systems with further health management capacity and those that offered more contextual health information. Two papers reported that participants wanted the system to provide reminders for upcoming appointments, remind them when to refill medications, and help them manage their bills and health status over time [17,28]. In 3 papers, participants wanted the system to provide lifestyle advice and tips or a dictionary of medical terms [17,22,29]. Participants in 2 papers wanted the system to provide diagnosis and prognosis [28,29].

Other participants requested features specific to medication such as warnings about medication interactions and the ability to make changes to their medication lists [30,29]. Hourcade et al (2011) [22] suggested that medication information and warnings should be layered from basic to advanced information [22]. Other desired features included ability to print information, access to complete medical records, having good technical support, and ability to take voice commands [21,29].

### Changes in Health Information Management and Provider Communication

Five papers described the impact of patient portals on health information management, focusing on increased access to records and improved storage of health information [10,17,20,21,24]. Zettel-Wattson and Tsukerman (2016) [17] explained that 90.6% of portal users (56/62) thought a portal helped them better manage health, and 89.7% (55/62) reported that health management tools allowed them to keep all of their records in one place. Additionally, 80.4% (50/62) explained that health information tools gave them a sense of control over their health.

In one paper, findings regarding older adult views on record access and management were mixed: 86% of participants (69/80) wanted access to their records in one place but did not necessarily want to be responsible for managing records, and 84% of participants (67/80) preferred that their records continue to be managed by primary care providers [20].

Papers also described changes in patient-provider communication. In one paper, participants expressed that having access to patient portals made them feel more prepared for emergencies and made visits with providers more efficient [24]. However, physicians thought that giving patients access to records may increase their worry [20], and some patients were concerned about a loss of face time with providers [21].

### Areas to Explore

Health literacy, defined as the ability to collect, interpret, and process basic health information [32], was mentioned in 4 papers as a barrier. However, these papers measured and defined health literacy differently [9,26,27], making it difficult to categorize health literacy as a barrier in this review but highlighting it as an area for future research. Of those papers that mentioned health literacy, one defined and measured health literacy by looking at participant questions related to the content of patient portals, particular diseases, and interpreting medical terminology [26]. This paper found that health literacy was a barrier for 29% of participants (11/38) who had questions about these issues [26]. Logue and Effken (2012) [27] defined and measured health literacy using the eHealth Literacy Scale (eHEALS) and criteria that looks specifically at the ability to identify, evaluate, and synthesize health information delivered electronically. They found that all three eHealth literacy indicators from the eHEALS were positively correlated with confidence in communicating with others on the Internet, ability to express oneself in writing, and using an Internet-based PHR. Taha et al (2014) [9] measured health numeracy or the ability to interpret health information reported as numbers. They found that 52.9% of their participants (27/51) correctly answered only 5 or fewer objective numeracy questions on an 11-question measure. However, on a Subjective Numeracy Scale, which measures perceived health numeracy, several participants gave themselves a high rating, indicating that many had overestimated their health numeracy skills [9].

### Design Suggestions

Several papers provided guidance about features and functions of patient portals and ePHRs [9,23,28]. At a basic level, these systems should provide health information, including medical history, test results, and medication information [28]. Information should be provided in a way that does not overwhelm the user [23,28]. Tools and aids were suggested to help users gain an understanding of health information and complete health management tasks [9]. Price et al (2013) [28] suggested that an ePHR should provide memory support to patients. For example, it should store a patient’s health history and help them remember daily tasks [28]. Khan et al (2010) [23] mentioned a need for clear communication between experts, designers, and patients regarding their understanding of personal health information. This would guard against the bias of one group impacting system design [23].

## Discussion

### Overview

With this review, we set out to identify and assess the evidence of barriers and facilitators to the use and adoption of patient portals and ePHRs by older adults. We also wanted to gain an understanding of older adults’ experiences with these systems and learn about the design recommendations resulting from study findings. Through our systematic review, we identified 2 barriers (privacy and security, and access and abilities) and 2 facilitators (technical assistance, and family and provider advice) to the use and adoption of patient portals and ePHRs. We also gained an understanding of older adults’ experiences with these systems, specifically perceived benefits, satisfaction, and desired features. Some of the papers did not present specific design recommendations, making it difficult to translate findings to improve the design of patient portals and ePHRs. We also found that some papers lacked a detailed description of patient portals or ePHRs; this is an issue because systems are not static and likely changed over time. Having a detailed description of the system would provide context to study results.

Overall, even though we were able to identify barriers and facilitators, the evidence lacked strength. There were several reasons for this, including the fact that many of the studies had a small sample size and were a convenience sample. In addition, our search results included a diversity of studies, making it difficult to draw firm conclusions related to our research questions.

It should also be noted that, throughout our analysis, we reported themes by grouping papers on ePHRs and patient portals together. It could be argued that the type of technology used (ePHR vs patient portal) would influence results related to user experience, barriers, and facilitators. When it came to barriers and facilitators, we noticed no clear trends in terms of concerns about privacy and security but found that the barrier of access to the Internet was more often mentioned in papers about patient portals [10,20,21], whereas facilitators were mostly mentioned in papers that focused on ePHRs [22,24,25,27]. However, this could be because there were very few papers focused on facilitators in general, and the majority of those that mentioned facilitators were also looking at initial use [17,19,24,25,27]. In contrast, the papers on patient portals that mentioned barriers were all formative in nature [10,20,21]. This difference in paper topic (formative vs initial use) may have accounted more for the patterns in results related to barriers and facilitators than the technology itself. In terms of user experience, there were no overall trends demonstrating differences between ePHRs and patient portals. However, log-in issues were reported only from formative papers involving patient portals [10,21], and suggestions for added features (discussed in detail under experience and design) came mainly from papers involving ePHRs [22,23,25,28,29].

On the basis of our review, we identified a need for more longitudinal evaluation of patient experience and use, more nuanced understanding of older adult subgroups, and further discussion of barriers and facilitators to inform design recommendations. There were 2 papers [19,24] that looked at older adult portal use through a cohort study design, examining log-in data and uses of the portal over the course of a year [19] and almost 3 years [24]. However, other papers examined average length of use of the portals. One paper and found that several participants used the portal for longer than a year [25] and in another paper several participants used the portal for an average of 3 years [17]. Further research with longitudinal studies could help to show how use evolves into adoption and why. It could also help to better identify barriers and facilitators to adoption of patient portals or ePHRs.

Papers used different approaches to evaluate patient portals or ePHRs. Although common themes emerged across papers, the variety of approaches made drawing conclusions difficult. It would be helpful to have more research on specific and widely used systems to produce results that are comparable and generalizable.

### Principal Findings

#### Barriers and Facilitators

Overall, it was more common for papers to describe barriers than facilitators to patient portal use. Concerns about privacy and security and lack of access or ability to use computers and technology were all commonly identified as barriers. These barriers are consistent with what has been identified in related literature. Some barriers were explained in more detail than others, and very few papers offered concrete solutions for addressing barriers, particularly among older adult populations.

Papers consistently described privacy and security issues. However, there were not many specific suggestions for making older adults feel secure, and there were no design suggestions from older adults about what would make them trust the security of a system.

Other papers more specific to privacy and security concerns found that although unauthorized access to records was an issue for older adults, it was also a concern for the general population [33]. In fact, older adults were significantly more willing than the general population to share health information with a provider [33]. Privacy and security concerns about patient portals are warranted, especially in today’s climate where breaches to data are often in current news. For example, in 2016, Molina Healthcare shut down its patient portal because of a security flaw that allowed patients to access other patient’s claims without authentication [34]. In 2017, there was a breach of UC Davis Health patient health records when an employee responded to a phishing email that allowed the hacker to access the employee’s emails and personal health information of patients. Fifteen thousand patients were impacted by this incident [35].

Authors of security-specific literature offered design suggestions to alleviate privacy concerns such as allowing patients to restrict access and sharing within a portal, and providing patients with an access log and list of any changes to medical information [33]. More research should be done to determine whether these and other design suggestions can work to mitigate security concerns, while still providing a positive user experience. Addressing security concerns could affect usability of a system. For example, users required to go through a 2-step log-in may perceive it as being cumbersome [36].

In patient portal research in general, there is recognition of systematic gaps in technology access and portal use [37-40]. Similar gaps in access have been identified in this literature review. Gordon and Hornbrook’s (2016) [19] paper was an exemplary publication with a large sample size that identified differences in portal use and technology access within subpopulations of older adults based on age and race and asked critical questions about physical ability. However, among the papers reviewed, there was not enough evidence to understand whether there are inequities in access to technology that in turn influence older adults’ portal use, skill, and quality of health care at a broader level. As noted by Kneale and Demiris (2017) [41], evaluations of patient portals often lack diversity or fail to report differences based on race, ethnicity, and gender. Generally, evaluations that report demographics conduct evaluations primarily with younger, white, non-Hispanic males who are highly educated [41]. Further evaluation of socioeconomic, racial, and gender disparities is necessary. Only a few papers drew explicit connections between access and its impact on perceived computer and Internet skills [10,26]. These papers generally did not examine the reasons behind computer anxiety or lack of confidence. Understanding and overcoming perceived barriers may be key to encouraging use and adoption of portals, but more research is necessary to identify why these perceived barriers exist.

A more in-depth discussion of facilitators, particularly among different cultural, social, and economic groups of older adults, may also be an important step toward creating a supportive system for older adults. Mention of facilitators in the literature is mainly limited to providing technical assistance [17,21,24,26]. Only Gordon and Hornbrook [19] offer suggestions for large-scale assistance programs, including user handbooks, a hotline, and workshops. Another facilitator described in the literature was provider advice. Although provider perspective was not the focus of our review, other studies suggest that provider EHR use has an impact on whether patients adopt portal technology [42,43]. Overall, additional research should focus on what facilitators are important to older adults and how these facilitators can be incorporated into the patient portal experience and implementation.

#### Health Literacy

Low health literacy and technology have been identified as barriers for adoption of patient portals among underserved adult populations [37,44], and privacy and technological concerns are common barriers to older adults adopting technology in general [45]. In this review, the papers varied in the way they defined and measured health literacy. One looked at health literacy by focusing on numeracy [9], another used eHEALS [27], and another measured health literacy by the number of questions that were asked about the content in the patient portal [26]. More research is needed to measure this barrier using a uniform method to identify how it affects portal use for older adults and to find design or implementation solutions that can be used to support health literacy among different subgroups of the older adult population.

#### Experience and Design

The papers in this review have used exploratory and evaluative methods to understand the factors that impact the use and experience of patient portals and ePHRs. However, there are opportunities to apply a design framework to developing patient portals and ePHRs. Nath and Sharp (2015) [46] proposed building on existing research methods, such as those that identify patient needs and preferences, using approaches such as user-centered design. Doing so will bridge the gap between needs and preferences and the design of a system. User-centered design is a process that aims to create usable systems that improve productivity, enhance user acceptance, reduce errors, and offer training and support. Human-centered design is based on the principle of actively involving users who have contextual knowledge of the tasks the system will be used for and the environment that the system will be used in. Human-centered design principles also include gaining an understanding of the tasks that the system will do, gaining early feedback from users through prototypes, and involving a multidisciplinary team [47].

Many of the papers reviewed identified barriers and facilitators to use and adoption. There were some that also gathered requirements for and input on system development. These findings can be used in the user-centered design process. There could be additional exploratory research done to gain an understanding of the user in context and the tasks they aim to complete. Including the user at the beginning of the process ensures that their needs are a part of the design process. Participatory design approaches have been used in this framework to engage and empower older adults in designing technology such as smart homes [48,49]. Using inclusive approaches can lead to unexpected discoveries of functions and features that are important to older adults.

Although studies in this review captured overall user experience, there is room for more exploration to better understand older adults’ experience with and use of patient portals and ePHRs. Research could focus on usability by learning about participants’ expectations and navigation of systems. This information could then provide designers with necessary feedback to make iterative improvements to particular systems. To understand what older adults need from patient portals and ePHRs, designers should consider including older adults in the design process.

This review looked across the user experience, examining both patient portals and ePHRs. However, these technologies do offer different experiences. The primary difference is that, as patient portals are tethered to the patient’s health record, patients do not need to manually enter their information, whereas ePHRs, which are not typically tethered to a patient’s health record, require patients to manually enter information. This distinction has impacted the user experience and resulted in some of the feedback about desired functionality of ePHRs that is solved by patient portals, such as limiting the amount of text entry, providing access to lab results, and the ability to contact providers. However, there were still some desired features that could be further investigated, such as reminders for appointments and medication refills, lifestyle tips, help managing claims, and voice commands. The differences between patient portals and ePHRs can perhaps also be seen as an impact of technology developing over time.

Considering that people are increasingly incorporating technology into their daily lives, desired features that provide contextual advice are a reasonable expectation. However, further research with older adults is needed to understand how patient portals or ePHRs could be integrated into older adults’ health management. In addition, researchers should consider relating their findings to the design of patient portals and ePHR systems. The recommendations could provide actionable changes and lead to opportunities to explore for potential features and functionality of the systems. For example, one desired feature mentioned in a paper was voice activation; patient portals could be paired with an intelligent personal assistant, such as the Amazon Echo, to increase convenience and access to health information.

Another consideration is for researchers and designers to think about the long-term adoption of these systems. Friedman and Nathan [49] proposed an approach called multi-lifespan information system design to challenge the short life cycle of a technology, which is usually 5 years. It asks researchers to think about the future of the technology, including its impact and how its use might change over time [49]. The method may be fitting for the design of patient portals and ePHRs because they are systems available for a wide range of people and may be used over lifetimes and generations.

### Limitations

The search terms for this systematic review were carefully chosen and aimed to draw a wide search. However, patient portals and ePHRs can be described differently, and some papers may have been missed. Our wide search also resulted in a diverse set of papers that presented challenges to drawing specific conclusions related to our research questions. Due to our focus on older adults, we eliminated papers that focused on provider perspectives as well as papers that focused on the health implications of patient portal implementation. We also excluded papers that were not in English, and so, we may have missed papers that were pertinent to our topic but in a different language. In addition, our key themes were determined based on a small number of papers. Even though our review included papers that analyzed patient portal and ePHR use among age groups other than older adults, we did not do a comparison between older adults and those other age groups. In addition, because of the large range of ages, 60 years and older, we did not distinguish the impact of age on the exposure to technology. Finally, our search criteria spanned over a 10-year period; it is important to recognize the constantly changing technology environment and the advances that have been made to patient portals and ePHRs over the 10-year span of time. These advances likely impacted the use and experience of participants across the studies that were reviewed.

### Conclusions

This review focused on understanding the barriers and facilitators to older adults’ use and adoption of patient portals and ePHRs. Across the studies there were 2 main barriers: (1) concerns about privacy and security and (2) access and ability to use technology and the Internet. The 2 main facilitators were receiving technical assistance with a patient portal or ePHR and receiving advice to use patient portals from family and providers.

In terms of older adults’ experience using patient portals and ePHRs, some papers indicated that patient portals and ePHRs helped older adults to better manage their health information. Older adults liked having a single place that they could access and archive their information. In some cases, older adults felt their communication with providers had improved because of their use of patient portals. Older adults also suggested improving patient portals and ePHRs to help them manage their health beyond record storage, for example, by providing diagnosis and prognosis.

Overall, this review demonstrated that there are a range of studies and methods to understand patient portal and ePHR use and experience among older adults. However, more research is needed to better understand and address barriers to patient portal and ePHR use and adoption by older adults. As many health care systems offer their patients a portal to their health information, there are opportunities for it to be an integral part in keeping patients informed about their health information and encouraging them to take an active role in their health care. This opportunity is especially great for the older adult population as it is expected to grow rapidly. In addition, evaluation of patient portal and ePHR systems should be continually done after they are launched to learn about the areas that are working and areas that could be improved. This is in line with the user-centered design process and communicates to users the organization’s commitment to deliver a positive user experience. Finally, the changing technology landscape should be considered in the design process to design a system that is flexible and would ease future transitions from legacy systems.

## Appendix

Multimedia Appendix 1 Final set of 17 papers.

Multimedia Appendix 2 Paper summaries.

Multimedia Appendix 3 Outcome of quality review.

## Abbreviations

AHRQ: Agency for Healthcare Research and Quality
eHEALS: eHealth Literacy Scale
EHR: electronic health record
ePHR: electronic personal health record
PHIMS: patient health information management system
PHR: personal health record
PRISMA: preferred reporting items for systematic reviews and meta-analyses
STARE-HI: Statement on the Reporting of Evaluation studies in Health Informatics
