# Supplementary material for: Patient Portal Use and Experience Among Older Adults: Systematic Review
Source: JMIR Med Inform. 2017 Oct 16;5(4):e38. doi: 10.2196/medinform.8092 (PMC5662789; doi:10.2196/medinform.8092)
Supplement: Multimedia Appendix 1 [file medinform_v5i4e38_app1.pdf]

Study	Goals and Results
Barron et al., 2014 [18]	Evaluates whether older adults with chronic illnesses and their caregivers have the ability to use a patient portal. 21.4% of patients and 11.8% of caregivers reported that the portal was moderately or very difficult to use. A majority of participants said they would continue using the patient portal.
Gordon and Hornbrook, [19]	Two studies of racial and age disparities in patient portal 2016 use, access to digital devices and preferences about interacting with the healthcare system. Seniors 70 years or older were less likely than younger seniors between 65-69 to have registered or signed in or used the patient portal features like secure messaging.
Haverhals et al., 2011 [30]	Identifies issues that PHAs could address among older adults and caregivers who are self-managing medication. Five key concerns include: finding information they can rely on, maintaining the independence to make medication decisions, taking too many medications, understanding difference between allopathic and alternative therapies, and coordinating information across multiple providers.
Hourcade et al., 2011[22]	Sessions with older adults to learn about their needs for a personal health records system focused on medication management. Older adults wanted more information and warnings about the medication they were taking but were confused by technical language. Expressed concerns about pharmaceutical companies accessing data.
Kerai et al., 2014 [20]	Evaluates patient views of Australia's national personal controlled electronic health record (PCEHR) concept, as opposed to evaluating the system through use. Patients were willing to adopt portals but identified changes in patient-provider communication as a concern. Patients were willing to share information with primary care providers, but not with other health professionals.
Khan et al., 2010 [23]	Six user studies of a web-based PHA, Colorado Care Tablet (CCT). Suggestions included: balance intuitive, health related images and informational text, require minimal input from the user, and communicate responsibilities for medication reconciliation between patients, providers, and caregivers and avoid translating miscommunication into system design.
Kim et al., 2009 [24]	Evaluated use of and satisfaction with Personal Health Information Management System (PHIMS) among a low income elderly population. Majority of participants were satisfied with the system and reported improved quality of health care. 92% shared PHIMS info with provider and felt more prepared for medical emergencies.
Lam et al., 2013 [25]	Compares older and younger adult attitudes toward electronic communication with providers. Both younger and older adults currently communicate with providers by phone but preferred to communicate by email. Messages sent, experiences with the system, and barriers were similar between younger and older adults.
Latulipe et al., 2015 [21]	Examining adoption of patient portals among older adults and caregivers four low-income counties. Benefits mentioned were convenience and archived health information. Main barriers were security and privacy. 15 of 36 non-users were not interested in adopting a portal, some felt too old to learn how to use a computer, Internet, or portal.

Lober et al, 2006 [26]	Descriptive study of low-income older adults' experience with a personal health information management system (PHIMS). Major barriers included: computer literacy, security, and the need for assistance with login and use.
Logue and Effken, 2012 [27]	Exploratory study on how factors in the Personal Health Records Adoption Model (PHRAM) would apply to older adults with chronic illnesses. Older adults reported access to computers but lack of access to/familiarity with the Internet. More chronic illnesses were associated with increased ePHR use.
Montelius et al., 2008 [31]	Online survey evaluating the Swedish online health management tool "My Dispensed Medications". Among older adults, getting control was selected as the most common reason for accessing the site. For younger age groups, curiosity was the main reason. Willingness to share information with family and physicians increased with age.
Price et al., 2013 [28]	Two studies comparing health information management needs of older and younger adults. Older and younger adults both had questions about specific symptoms and medications, and were more likely to keep information on procedures, test results, and immunizations than other health materials. Privacy was a bigger barrier to adoption than usability. Concerns about backing up information and identity theft also expressed.
Sack et al., 2011 [29]	Looked at relative costs and benefits of web and mobile-based ePHRs for older adults. More costs than benefits mentioned for the website, and for the mobile site the costs and benefits were even. Older adults wanted warnings about medication interaction, and information about their diagnosis and prognosis if applicable.
Taha et al., 2014 [9]	Evaluated older adult use of patient portals to complete health-management tasks. Most participants had health literacy scores in the "adequate" range, and participants overestimated their ability to comprehend numeric information. 11.8% of participants completed simple tasks in a portal and none could complete the complex tasks. 86.3% would use the patient portal if it was available from their doctor.
Turner et al., 2015 [10]	Semi-structured interviews addressing older adult use of patient portals. Positive features of portals included: easy access to health information, direct communication with providers, ease of scheduling and a decrease in physical record keeping. Negative comments included security concerns, lack of desire to use portals, and difficulty using computers.
Zettel-Watson and Tsukerman, 2016 [17]	Compared users and non-users of online health management tools. Participants most frequently used tools to check lab results, store records, or manage chronic diseases. Users wanted to perform more functions, such as scheduling appointments or viewing more detailed health information. Older age was associated with more willingness to share online health information with adult children.
