# Supplementary material for: Patient Portal Use and Experience Among Older Adults: Systematic Review
Source: JMIR Med Inform. 2017 Oct 16;5(4):e38. doi: 10.2196/medinform.8092 (PMC5662789; doi:10.2196/medinform.8092)
Supplement: Multimedia Appendix 2 [file medinform_v5i4e38_app2.pdf]

Author	Focus of Study	Study design or duration	Systems evaluated	Participants	Quality rating
Barron et al., 2014 [18]	Formative; focuses on information gathering for design and system development<br><br>Does not distinguish between short-term users and adopters	Qualitative; interviews and cognitive walkthrough<br><br>One-time interview and walk-through	Patient portal; system developed at Johns Hopkins University geriatric medical practice	N=33; n=14 older adults, n=19 caregivers<br><br>Older adult average age: 78.6 years<br><br>Gender: 50% (7/14) female	5
Gordon and Hornbrook, 2016 [19]	Use; restricts analysis to those who have logged onto a portal and viewed a lab test or used a prescription refill at least once in a calendar year<br><br>Does not distinguish between short-term users and adopters	Quantitative; patient portal use study and survey Cross-sectional	Patient portal; evaluates a health plan portal	N=231,082 older adults;<br>n=2602 survey respondents<br><br>Average age: not reported; survey respondent age brackets: 23.48% (611/2602 65-69 years), 43.7% (1137/2602 70-74 years), and 32.78% (853/2602 75-79 years)<br><br>Gender: 54.07% female (1407/2602)	6
Haverhals et al., 2011 [30]	Formative; focuses on information gathering for design<br><br>Does not distinguish between short-term users and adopters	Qualitative; interviews and focus groups<br><br>One-time interviews and focus groups	N/A; identifying health information management issues to address in development of personally managed health applications generally	N=34; n=32 older adults, n=2 caregivers<br><br>Older adult average age: 82 years<br><br>Gender: 60% (9/15) female	6

Hourcade et al., 2011 [22]	Formative; focuses on information gathering for design and system development<br><br>Does not distinguish between short-term users and adopters	Qualitative; design sessions<br><br>Twelve 1-hour sessions over 4 weeks	ePHR; Gathering design recommendations for development of a personal health record focused on medication management	N=16 older adults, residents at a retirement community;<br><br>**group 1** n= 8 residents at a retirement community median age: 78 years;<br><br>**group 2** n= 8 different residents at same retirement community median age: 75 years<br><br>Gender: 50% (8/16) female	7
Kerai et al., 2014 [20]	Formative; looks at perceptions about initial acceptance of a portal<br><br>Does not distinguish between short-term users and adopters	Quantitative; questionnaire<br><br>Cross-sectional	Patient Portal; Australia's personally controlled electronic health record, which is tethered to an electronic medical record but has patient controlled sharing and visibility	N=80 older adults<br><br>Older adult median age: 71 years<br><br>Gender: 62% (50/80) female	8
Khan et al., 2010 [23]	Formative; focuses on information gathering for design and system development.<br><br>Does not distinguish between short-term users and adopters	Qualitative; user study<br><br>One-time user studies	ePHR; evaluated prototypes and a functional system—the Colorado Care Tablet	N=31; n=22 older adults, n=9 caregivers<br><br>Older adult average age: 76.4 years<br><br>Gender: not reported	8
Kim et al., 2009 [24]	Use and Adoption; compares frequency of use and patterns of	Mixed study; questionnaire and system use<br><br>33-month	ePHR; PHIMS	N=70 residents; n=44 older adults; residents of a low income housing complex	6

	use over a 33-month study period	longitudinal study		Older adult average age: 63.1 years<br><br>Gender: 71% (50/70) female	
Lam et al., 2013 [25]	Adoption and Use; Survey measured length of use as less than a few months, a few months to 1 year, and more than 1 year.<br><br>Majority of respondents used the system a few months to 1 year	Mixed; survey; comparison of older and younger adults<br><br>Cross-sectional	ePHR; untethered messaging system for talking with a medical team called Vision Tree	N=372 total; n=145 older adults<br><br>Older adult average age: 74.9 years<br><br>Gender: not reported	7
Latulipe et al., 2015 [21]	Formative; focuses on information gathering for design<br><br>Does not distinguish between short-term users and adopters	Qualitative<br><br>One-time interview	Patient portal; no specific system evaluated	N=52; n=36 patients, n=16 caregivers, n=23 patients over age 65 years<br><br>Older adult average age: not reported<br><br>Gender: 57% (13/23) female	7
Lober et al., 2006 [26]	Use; examines barriers to initial use and participants' feelings about the personal health information management system (PHIMS)<br><br>Does not distinguish between short-term users and adopters	Mixed study; observations and survey 6-month longitudinal study	Electronic personal health record (ePHR); PHIMS	N=38 older adults; residents of a low income housing complex<br><br>Older adult average age: 69 years<br>Gender: 82% (31/38) female	5

Logue and Effken, 2012 [27]	Use; looks at factors influencing initial use<br><br>Does not distinguish between short-term users and adopters	Quantitative; descriptive survey<br><br>Cross-sectional	ePHRs; no specific system evaluated	N=38 older adults; residents of two retirement communities<br><br>Older adult average age: 77 years<br><br>Gender: 71% (25/35) female	7
Montelius et al., 2008 [31]	Use; examines reasons for initial use and feelings about the website<br><br>Does not distinguish between short-term users and adopters	Quantitative; Web-based survey; comparison of older and younger adults<br><br>Cross-sectional	Not applicable (N/A); Swedish Government operated, Webbased medication list: “My Dispensed Medications”	N=1716 total; n= 225 older adults<br><br>Older adult average age: not reported 13.11% (225/1716) of total are 65 years or older<br><br>Gender: 78.67% (177/225) female	7
Price et al., 2013 [28]	Formative; focuses on information gathering for design<br><br>Does not distinguish between short-term users and adopters	Qualitative; diary method and interviews; comparison of older and younger adults<br><br>2-week diary; one-time interview	ePHR; no specific system evaluated	N=72;<br>**study 1:** n= 24 older adults; average age: 72 years<br><br>**study 2:** n=12 older adults; average age:74<br><br>Some of the participants in the first study were also in the second study.<br><br>Gender: not reported	8
Sack et al., 2011 [29]	Use; examines reasons for initial use and the perceived cost and benefits of systems<br><br>Does not distinguish between short-term users and adopters.	Qualitative; focus groups<br><br>One-time focus group	ePHR; comparing Google Health accessed through a Web-based and mobile app	N=26 older adults<br><br>Older adult average age: 71 years<br><br>Gender: not reported	6

Taha et al., 2014 [9]	Formative; focuses on information gathering for design<br><br>Does not distinguish between short-term users and adopters	Mixed study; questionnaires and user study cross-sectional survey and one-time evaluation	Patient portal; simulated system called CREATE	N=51 older adults<br><br>Older adult average age: 69.31 years<br><br>Gender: 60.8% (31/51) female	8
Turner et al., 2015 [10]	Formative; focuses on information gathering for design<br><br>Does not distinguish between short-term users and adopters	Qualitative; comparison of portal users to portal nonusers<br><br>One-time interview	Patient portal; no specific system evaluated	N=74 older adults<br><br>**Portal user:** average age: 76 years ;<br>**Portal nonuser:** average age: 78 years<br><br>Gender: Portal user 87% (13/15) female; Portal nonuser 59% (35/59) female	7
Zettel-Watson and Tsukerman, 2016 [17]	Adoption and use; measured length of time using system, which ranged from 1 month to 10 years, with an average of 3 years	Quantitative; survey; comparison of portal users to portal nonusers<br><br>Cross-sectional	Looks at Web-based health management tools and does not distinguish between ePHR and patient portal;<br>no specific system evaluated	N=166 older adults; portal users n=62, portal nonusers n=104<br><br>average age:<br>**portal user:** 68.5 years;<br>**portal nonuser:** 72.2 years<br><br>Gender:<br>portal user 72.6% (45/62) female;<br>portal nonuser 55.9% (58/104) female	6

References

18. Barron J, Bedra M, Wood J, Finkelstein J. Exploring three perspectives on feasibility of a patient portal for older adults. Proc 12th International Conference on Informatics, Management, and Technology in Healthcare, ICIMTH, Athens, Greece 2014; (202): 181-184. PMID: 25000046

19. Gordon NP, Hornbrook MC. Differences in access to and preferences for using patient portals and other eHealth technologies based on race, ethnicity and age: a database and survey study of seniors in a large health plan. J Med Internet Res 2016; 18(3): e50. PMID: 26944212

30. Haverhals LM, Lee CA, Siek KA, Darr CA, Linnebur SA, Ruscin JM, Ross SE. Older adults with multimorbidity: medication management processes and design implications for personal health applications. J Med Internet Res 2011; 13(2): e44. PMID: 21715286

22. Hourcade JP, Chrischilles EA, Gryzlak BM, Hanson BM, Dunbar DE, Eichmann DA, Lorentzen RR. Design Lessons for Older Adult Personal Health Records Software from Older Adults. In: Stephanidis C, editors. Universal Access in Human-Computer Interaction. Users Diversity UAHCI 2011 Lecture Notes in Computer Science. Springer, Berlin, Heidelberg; 2011. vol 6766. ISBN: 978-3-642-21662-6.

20. Kerai P, Wood P, Martin M. A pilot study on the views of elderly regional Australians of personally controlled electronic health records. Int J Med Inform 2014; 83(3): 201-209. PMID: 24382474

23. Khan DU, Siek KA, Meyers J, Haverhals LM, Cali S, Ross SE. Designing a personal health application for older adults to manage medications. IHI’10 Proceedings of the 1st ACM International Health Informatics Symposium; 2010 November 11-12; Arlington, Virginia. New York: ACM; 2010. DOI: 10.1145/1882992.1883124

24. Kim EH, Stolyar A, Lober WB, Herbaugh AL, Shinstrom SE, Zierler BK, Soh CB, Kim Y. Challenges to using an electronic personal health record by a low-income elderly population. J Med Internet Res 2009;11(4): e44. PMID: 19861298

25. Lam R, Lin VS, Senelick WS, Tran HP, Moore AA, Koretz B. Older adult consumers' attitudes and preferences on electronic patient-physician messaging. Am J Manag Care 2013; 19(10): eSP7- eSP11. PMID: 24511886

21. Latulipe C, Miller DP, Gatto A, Quandt SA, Nguyen HT, Bertoni AG, Smith A, Arcury TA. Design considerations for patient portal adoption by low-income, older adults. Proceedings of the 33rd Annual ACM Conference on Human Factors in Computing Systems; 2015 April 18-23; 3859-3868. PMID: 27077140

26. Lober WB, Zierler B, Herbaugh A, Shinstrom SE, Stolyar A, Kim EH, Kim Y. Barriers to the use of a personal health record by an elderly population. Proc AMIA Annual Symp 2006; 514-518. PMID: 17238394

27. Logue MD, Effken JA. An exploratory study of the personal health records adoption model in the older adult with chronic illness. Inform Prim Care 2012; 20 (3): 151-169. PMID: 23710840

31. Montelius E, Astrand B, Hovstadius B, Petersson G. Individuals appreciate having their medication record on the web: a survey of national attitudes to a national pharmacy register. J Med Internet Res 2008; 10(4): e35. PMID: 19000978

28. Price MM, Pak R, Muller H, Stronge A. Older adults' perceptions of usefulness of personal health records. Universal Access in the Information Society 2013; 12(2): 191-204. DOI: 10.1007/s10209-0120275-y

29. Sack O, Pak R, Ziefle, M. Older adults' perception of costs and benefits of web-based and mobile PHR technologies: a focus group approach. Proceedings of the Quality in e-Health - 7th Conference of the Workgroup Human-Computer Interaction and Usability Engineering of the Austrian Computer Society USAB; 2011 November 25-26; Graz, Austria. Berlin: Springer; 2011. DOI: 10.1007/978-3-642-25364-5_52.

9. Taha J, Sharit J, Czaja SJ. The impact of numeracy ability and technology skills on older adults' performance of health management tasks using a patient portal. Journal Appl Gerontol 2014; 33(4): 416436. PMID: 24781964

10. Turner AM, Osterhage K, Hartzler A, Joe J, Lin L, Kanagat N, Demiris G. Use of patient portals for personal health information management: the older adult perspective. Proc AMIA Symp 2015; 1234-41. [Medline]

17. Zettel-Watson L, Tsukerman, D. Adoption of online health management tools among healthy older adults: an exploratory study. Health Informatics J 2016; 22(2): 171-183. PMID: 25149210
