# Supplementary material for: Patient Portal Use and Experience Among Older Adults: Systematic Review
Source: JMIR Med Inform. 2017 Oct 16;5(4):e38. doi: 10.2196/medinform.8092 (PMC5662789; doi:10.2196/medinform.8092)
Supplement: Multimedia Appendix 3 [file medinform_v5i4e38_app3.pdf]

Article	Interpret the data and an answer to the study question	Description of the outcome measure/evaluation criteria	Provides a description of system and its functionalities	Provide clear description of how results impact design recommendations	**Total**
Lober et al, 2006 [26]	2	1	2	0	**5**
Montelius et al., 2008 [31]	2	2	2	1	**7**
Kim et al., 2009 [24]	2	2	2	0	**6**
Khan et al., 2010 [23]	2	2	2	2	**8**
Haverhals et al., 2011 [30]	2	1	1	2	**6**
Hourcade et al., 2011 [22]	2	2	1	2	**7**
Sack et al., 2011 [29]	2	2	1	1	**6**
Logue and Effken, 2012 [27]	2	2	2	1	**7**
Lam et al., 2013 [25]	2	2	2	1	**7**
Price et al., 2013 [28]	2	2	2	2	**8**
Barron et al., 2014 [18]	2	1	1	1	**5**
Kerai et al., 2014	2	2	2	2	**8**

[20]					
Taha et al., 2014 [9]	2	2	2	2	**8**
Latulipe et al., 2015 [21]	2	2	1	2	**7**
Turner et al., 2015 [10]	2	2	2	1	**7**
Gordon and Hornbrook, 2016 [19]	2	2	1	1	**6**
Zettel-Watson and Tsukerman, 2016 [17]	2	2	1	1	**6**
